# Self-reactive CD4^+^ T cells activated during viral-induced demyelination do not prevent clinical recovery

**DOI:** 10.1186/s12974-015-0426-1

**Published:** 2015-11-11

**Authors:** Carine Savarin, Cornelia C. Bergmann, Melanie Gaignage, Stephen A. Stohlman

**Affiliations:** Department of Neurosciences NC-30, Lerner Research Institute, The Cleveland Clinic Foundation, 9500 Euclid Avenue, Cleveland, OH 44195 USA; Present address: Unit of Experimental Medicine, de Duve Institute, Universite Catholique de Louvain, Brussels, Belgium

**Keywords:** Viral infection, Central nervous system, CD4 T cells, Demyelination, Autoimmunity, Antigen-presenting cells

## Abstract

**Background:**

Microbial infections have been implicated in initiating and enhancing severity of autoimmune diseases including the demyelinating disease multiple sclerosis (MS). Nevertheless, the incidence of both acute and persisting viral infections without evidence of autoimmune sequelae suggests that this process is well controlled. The conditions promoting or stemming self-reactive (SR) T cells following viral-induced tissue damage thus need to be better defined. Using a non-fatal viral mouse model of encephalomyelitis associated with demyelination and disability, yet ultimate clinical improvement, this study set out to monitor uptake and presentation of endogenous myelin antigens, as well as induction and fate of SR T cells.

**Methods:**

Activation and central nervous system (CNS) recruitment of myelin-specific CD4 T cells was analyzed by flow cytometry during encephalomyelitis induced by a glia tropic murine coronavirus. Potential antigen-presenting cells (APC) ingesting myelin were characterized by flow cytometry and their ability to activate SR T cells tested by co-culture with carboxyfluorescein succinimidyl ester (CFSE)-labeled myelin-specific CD4 T cells. Endogenous SR T cell kinetics was analyzed within both cervical lymph nodes and CNS by Enzyme-Linked ImmunoSpot (ELISPOT) following viral infection.

**Results:**

The data demonstrate the presence of APC capable of activating SR T cells in both draining lymph nodes and the CNS temporally correlating with overt demyelination. While both the CNS-infiltrating myeloid population and microglia ingested myelin, only CNS-infiltrating APC were capable of presenting endogenous myelin antigen to SR T cells ex vivo. Finally, SR T cell activation from the endogenous T cell repertoire was most notable when infectious virus was controlled and paralleled myelin damage. Although SR T cell accumulation peaked in the persistently infected CNS during maximal demyelination, they were not preferentially retained. Their gradual decline, despite ongoing demyelination, suggested minimal re-stimulation and pathogenic function in vivo consistent with the lack of autoimmune symptoms.

**Conclusions:**

The results demonstrate the potential for CNS tissue destruction to induce and recruit SR T cells to the injury site and support a host suppressive mechanism limiting development of autoimmunity.

## Background

Multiple sclerosis (MS) is an autoimmune disease of the central nervous system (CNS) characterized by demyelination, axonal loss, and increasing disability [1, 2]. While the etiology of MS remains unknown, various genetic and environmental factors have been associated with MS pathogenesis [3–5]. Among the environmental cues, viral infections, most prominently Epstein-Barr virus and human herpes virus type 6, have been linked to initiation or progression of disease [4, 6–9]; however, their causal nature remains unclear. Anti-viral antibodies and viral antigens have been detected in brain and cerebrospinal fluid of MS patients [10–12]. Nevertheless, active virus replication has yet to be demonstrated in the CNS of MS patients. By contrast, infections have even been proposed to limit autoimmunity [13]. Indeed, the decreased incidence of infectious diseases in the past decades inversely correlates with an increased incidence of autoimmune diseases, including MS [14]. For example, viral infection in an animal model of type I diabetes prevents disease development [15, 16]. Similarly, mice bred under conventional conditions are less susceptible to diabetes than mice bred in a pathogen-free environment [14], supporting the concept that microbiome burden may reduce the incidence of autoimmunity [17]. Therefore, the role of viral infections in autoimmune diseases is complex and remains largely elusive.

Activation of self-reactive (SR) immune cells by epitope spreading, molecular mimicry, cryptic antigen, and bystander activation have all been proposed to explain how viral infections may ultimately result in autoimmunity [18, 19]. This is exemplified by the biphasic disease induced by Theiler’s murine encephalomyelitis virus (TMEV) [20]. Minimal clinical deficits appear during the acute phase of TMEV infection, characterized by neuronal infection. The protective anti-viral immune response fails to completely eliminate infectious virus, resulting in chronic infection of microglia and macrophages [21], ascending paralysis and progressive demyelination [22]. Whereas initial tissue destruction occurs as a bystander effect of the anti-viral response, paralysis and demyelination are associated with autoimmunity mediated by SR T cells activated via epitope spreading during chronic infection [18, 22]. TMEV infection thus provides a paradigm for autoimmunity associated with chronic infection and sustained inflammation. By contrast, demyelination induced by infections with the neurotropic mouse hepatitis virus (MHV) strain JHM (JHMV) or the related dual hepato- and neurotropic strain MHV-A59 suggests that persistent CNS infections, even those associated with myelin destruction, do not necessarily result in clinically apparent CNS autoimmune disease. Although a vigorous CD8^+^ T cell- and Th1-dominated immune anti-viral response eliminates infectious virus [23], viral antigen and RNA persist within the CNS without evidence of active virus replication [24, 25]. Demyelination requires both infection of oligodendrocytes and adaptive immunity and is initiated by T cell-mediated virus control. Similar to chronic TMEV infection, persistent MHV infections are associated with ongoing demyelination. However, following acute viral-induced tissue injury, sustained demyelination is balanced by myelin repair, associated with clinical improvement [23, 26, 27]. The absence of disease progression during chronic MHV infection suggests that autoimmune responses are either not initiated or suppressed. Nevertheless, during acute MHV-A59 infection, sufficient myelin-derived self-antigen (Ag) is presented in cervical lymph nodes (CLN) to support proliferation of exogenously added SR T cells. However, little or no SR T cell proliferation occurred within the CNS, suggesting that T cells activated in CLN during acute infection migrate to the CNS but are unable to promote a local autoimmune response. The present study set out to determine whether Ag-presenting cells (APC) in the CNS support SR T cell proliferation and whether endogenous SR T cells are induced during JHMV-induced demyelination.

The data demonstrate that SR T cell activation is Ag-driven and mediated by a population of CD11b^+^ APC present within both the CLN- and the CNS-infiltrating cells. Despite myelin uptake and expression of major histocompatibility complex (MHC) class II molecules, microglia do not support SR T cell activation. Importantly, the kinetics of CD11b^+^ cell-mediated SR T cell activation parallels demyelination and correlated with myelin-specific T cells derived from the endogenous host T cell repertoire. Nevertheless, despite sustained demyelination and CNS inflammation, the SR T cell response declined during viral persistence, suggesting that chronic JHMV infection establishes an environment which supports ongoing clinical improvement and regulates autoimmune responses.

## Methods

### Mice

Wild-type (Wt) C57BL/6 mice were purchased from the National Cancer Institute (Frederick, MS, USA). Myelin oligodendrocyte glycoprotein (MOG)-specific T cell receptor (TCR) transgenic (2D2) mice expressing the congenic marker CD90.1 [28] were obtained from Dr. V.J. Kuchroo (Harvard University, Boston, MA). Ovalbumine (OVA)-specific TCR transgenic (OT-II) mice expressing the congenic marker Ly5.1 were provided by Dr. B. Min (Cleveland Clinic, Cleveland, OH). Mice expressing the green fluorescent protein (GFP) under the myelin proteolipid protein (PLP) promoter (PLP-GFP) [29] were obtained from Dr. W.B. Macklin (University of Colorado, Denver, USA). All transgenic mice were bred and maintained at the Biological Resources Unit of the Cleveland Clinic Lerner Research Institute under sterile conditions. All procedures were performed in compliance with the Cleveland Clinic Institutional Animal Care and Use Committee approved protocol number 2013-1131.

### Peptides

Peptides were obtained from Bio-Synthesis (Lewisville, TX, USA) and included M^133–147^ (GTVYVRPIIEDYHT), MOG^35–55^ (MEVGWYRSPFSRVVHLYRNGK), and MBP^60–80^ (SHHAARTTHYGSLPQKSQR).

### Virus

Mice were infected between 6 and 7 weeks of age in the left hemisphere with 1000 PFU of the glia tropic JHMV neutralizing monoclonal antibody (mAb)-derived 2.2v-1 variant [30]. Mice were assessed daily for clinical disease severity according to the following scale: 0, healthy; 1, hunched back and ruffled fur; 2, partial hind limb paralysis or inability to maintain the upright position; 3, complete hind limb paralysis; and 4, moribund or dead.

### Isolation of mononuclear cells

Anesthetized mice were perfused with phosphate-buffered saline (PBS). Single-cell suspension was prepared from the CLN by mechanical disruption through a 70-μm cell strainer in RPMI 1640 medium containing 25 mM HEPES (pH 7.2) supplemented with 10 % fetal calf serum (FCS). Brain and spinal cord were homogenized using a TenBroeck tissue grinder. Tissue homogenates were adjusted to 30 % Percoll (Pharmacia, Uppsala, Sweden). A 1-ml 70 % Percoll underlay was added prior to centrifugation at 850 g for 30 min at 4 °C. CNS-derived cells were recovered from the 30–70 % interface, washed, and resuspended in Roswell Park Memorial Institute (RPMI) 1640 medium 10 % FCS. Splenocytes were isolated from naïve mice by mechanical disruption and red blood cells eliminated using Gey’s solution.

### Enzyme-Linked ImmunoSpot assay

Ninety-six-well filtration plates (Millipore, Billerica, MA, USA) were coated overnight at 4 °C with anti-interferon-γ (IFN-γ) capture Ab (10 μg/ml; BD Biosciences, San Diego, CA, USA). Serial dilutions of CLN- and CNS-derived mononuclear cells pooled from a minimum of eight mice per time point were cultured in triplicate in RPMI complete (RPMI 1640 medium containing 2 mM L-glutamine, non-essential amino acid, 1 mM sodium pyruvate, 25 μg/ml gentamicin, and 5 × 10^−5^ M 2-mercaptoethanol) supplement with 10 % FCS in the presence of irradiated splenocytes (2.5 × 10^5^ cells/well) pre-incubated with or without 1 μM peptide for 2 h at 37 °C. After 36 h at 37 °C, spots were visualized by sequential incubation with biotinylated anti-IFN-γ mAb (5 μg/ml; BD Biosciences) overnight at 4 °C, horseradish peroxidase-conjugated streptavidin (BD Biosciences) for 1 h at room temperature, and 3,3′-diaminobenzidine substrate (Sigma Aldrich, St. Louis, MO, USA). Spots were counted using a CTL ImmunoSpot analyzer (Cellular Technology Ltd., Shaker Heights, OH, USA). Spots detected in wells with no stimulation (no peptide) were subtracted and results presented as the number of IFN-γ-secreting cells normalized to 10^6^ input cells.

### Flow cytometry

Non-specific binding was blocked with mouse serum and anti-mouse FcγIII/II mAb for 15 min on ice. Cells were stained for surface markers for 30 min on ice using fluorescein isothiocyanate (FITC), phycoerythrin (PE), peridinin chlorophyll protein complex (PerCP), or allophycocyanin (APC)-conjugated mAb (all from BD Biosciences) specific for CD45 (clone Ly-5), CD4 (clone GK1.5), CD8 (clone 53-6.7), CD25 (clone PC61), CD44 (clone IM7), CD45.1 (clone A20), CD90.1 (clone OX.7), CD11b (clone M1/70), CD80 (clone 16-10A1), and CD86 (clone GL-1). Cells were washed twice in FACS buffer (PBS, 1 % bovine serum albumin (BSA)) prior analysis. For proliferation study, bromodeoxyuridine (BrdU, 1 mg/mouse) (BD Biosciences) was injected i.p. 24 h prior to sacrifice. Mononuclear cells from CLN and CNS were prepared as described above, stained for surface markers followed by intracellular BrdU according to the manufacturer’s instructions using the FITC BrdU flow kit (BD Biosciences). Data were analyzed using a FACSCalibur flow cytometer (BD Biosciences) and FlowJo software (Tree Star Inc., Ashland, OR, USA).

### Isolation of naïve CD4^+^ T cells and adoptive transfer

CD4^+^ T cells were enriched from naïve 2D2 (TCR transgenic mice specific for MOG^35-55^) or OT-II (TCR transgenic mice specific for OVA) mice by negative selection using the CD4^+^ T cell isolation kit II (Miltenyi Biotec Inc., Auburn, CA, USA), according to the manufacturer instructions. Naïve (CD25^−^CD44^lo^) CD4^+^ T cells were then purified by cell sorting (FACSAria, BD Biosciences). Sub-lethally irradiated (450 rad) Wt recipients received equal numbers (10^6^ cells) of purified naïve OT-II and 2D2 CD4^+^ T cells by intravenous injections and were challenged with virus 2 weeks after adoptive transfer.

### Isolation of antigen-presenting cells and proliferation analysis

CLN, brains, and spinal cords were isolated at various times post-infection (p.i.) from PBS-perfused mice. CLN were mechanically disrupted through a 70-μm cell strainer as described above, whereas brains and spinal cords were finely minced with a razor blade. Suspensions were digested in RPMI 1640 medium containing 10 % FCS, 0.5 % collagenase (100 mg/ml) (Roche, Basel, Switzerland), and 1 % DNase I (1 mg/ml) (Sigma Aldrich) for 40 min at 37 °C. Collagenase was then inactivated by addition of 1 % 0.1 M EDTA for 5 min at 37 °C. Following centrifugation at 400 g for 7 min at 4 °C, CLN-derived cells were directly resuspended in FACS buffer for staining, whereas CNS-derived cells were isolated using Percoll gradients prior to staining as described above. CLN-derived CD11b^−^ and CD11b^+^ cells, CNS-infiltrating CD45^hi^CD11b^+^, microglia (CD45^lo^CD11b^+^), and CD45^hi^CD11b^−^ cells were purified using a cell sorter (FACSAria) and resuspended in RPMI complete 10 % FCS. Naïve CD4^+^ T cells were purified from splenocytes of naïve 2D2 mice using the CD4^+^CD62L^+^ T cell isolation kit II (Miltenyi Biotec) according to the manufacturer instructions. Naïve 2D2 CD4^+^ T cells were then stained with carboxyfluorescein succinimidyl ester (CFSE, 1.25 μM) (Molecular Probes, Carlsbad, CA, USA) and cultured in a 96-well plate in the presence of CLN-derived CD11b^−^ or CD11b^+^ cells, CNS-derived CD11b^−^ or CD11b^+^ cells, or microglia (T cells/APC ratio 1:1) in RPMI complete 10 % FCS for 4 days at 37 °C. Rat anti-MHC class II blocking mAb (clone M5/114) (Abcam, Cambridge, MA) or MOG^35-55^ peptide (10 μM) were added at the initiation of the cultures for some experiments. T cell proliferation was assessed by measuring the percentage of CFSE dilution by flow cytometry.

### Immunofluorescence

Mice were perfused with ice-cold PBS followed by 4 % paraformaldehyde (PFA). Spinal cords were dissected, fixed for 1 h in 4 % PFA at 4 °C, and then incubated with sucrose gradients as follows: 30 min with 15 % sucrose at room temperature, 30 min with 20 % sucrose at 4 °C, and, finally, overnight with 30 % sucrose at 4 °C. Tissues were stored in cryoprotection solution until preparation of 30-μm microtome sections. After antigen retrieval with 0.01 M sodium citrate buffer pH 6.0, sections were treated with 1 % Triton X-100 for 30 min, treated with blocking solution (PBS, 1 % BSA, 10 % normal goat serum) for 30 min, and stained with rabbit anti-mouse Iba1 (Wako, Richmond, VA) and mouse anti-mouse PLP (Millipore) primary mAbs overnight at 4 °C. Alexa Fluor 488 goat anti-rabbit (Invitrogen, Carlsbad, CA) and Alexa Fluor 594 goat anti-mouse (Invitrogen) were added for 1 h at room temperature. Sections were mounted with Vectashield mounting medium with 4′-6-diamidino-2-phenylindole (DAPI) (Vector Laboratories) and analyzed using a Leica TCS confocal microscope.

### Data analysis

Results represent the mean ± SEM and are plotted using GraphPad Prism software. Statistics were calculated using a two-tailed unpaired Student’s *t* test, ANOVA with Bonferroni post-test, and Dunn’s multiple comparison test, and *p* values <0.05 were considered statistically significant.

## Results

### Activation and CNS recruitment of SR CD4^+^ T cells

**Fig. 1:**
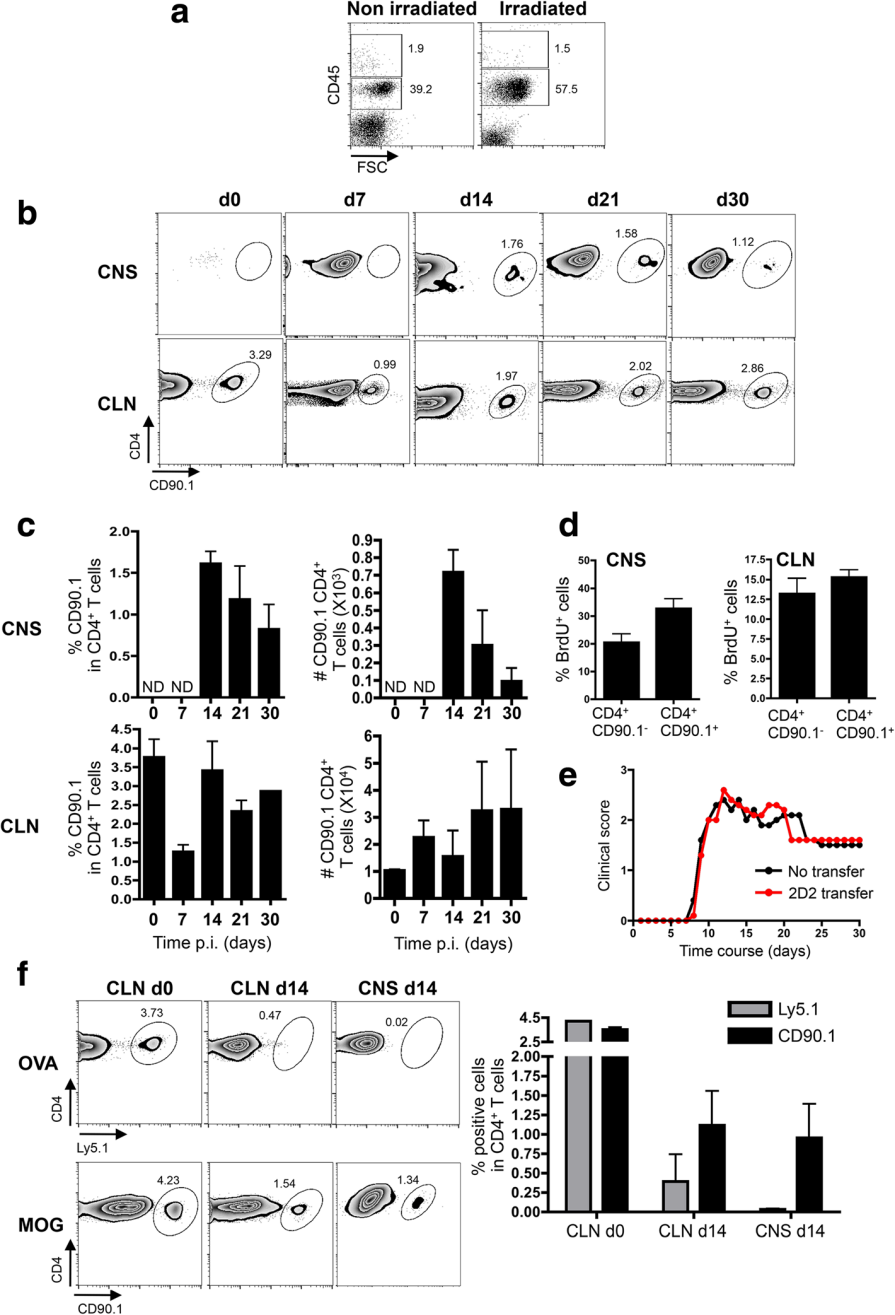
Peripheral activation and CNS recruitment of self-reactive CD4^+^ T cells is myelin driven. **a** Irradiated Wt mice received 1 × 10^6^ naïve MOG-specific 2D2 (CD90.1^+^) CD4^+^ T cells i.v. Two weeks post-transfer and prior infection, CD45^hi^ cells within the CNS were analyzed by flow cytometry and compared to age-matched non-irradiated Wt mice. **b** Representative FACS density plots of 2D2 cells within CD4^+^ T cells isolated from the CNS and CLN at days 0, 7, 14, 21, and 30 p.i. **c** Frequency and absolute number of 2D2 cells within CD4^+^ T cells in the CNS and CLN at days 0, 7, 14, 21, and 30 p.i. Data represent the mean of three individual mice per time point. **d** Frequencies of proliferating host CD90.1^−^CD4^+^ and transferred CD90.1^+^CD4^+^ T cells within the CNS and CLN, characterized by BrdU incorporation and analyzed by flow cytometry. Data represent the mean ± SEM of three individual mice. **e** Mean clinical scores following JHMV infection of WT mice without transfer (*n* = 15) or following 2D2 T cell transfer (*n* = 32). **f** Irradiated Wt mice received 1 × 10^6^ MOG-specific 2D2 (CD90.1^+^) CD4^+^ T cells and an equal number of OVA-specific OT-II (Ly5.1^+^) CD4^+^ T cells. Percentages of CD90.1^+^ 2D2 and Ly5.1^+^ OT-II cells within the CLN before infection (*d0*) and in both the CLN and CNS at day 14 p.i. *Bar graphs* represent the mean ± SEM of two separate experiments

Infection with the MHV-A59 strain suggested that acute encephalomyelitis provides a milieu capable of supporting proliferation of transferred MOG-specific T cell receptor (TCR) transgenic T cells within the CLN [31]. However, neither their reactivation within the CNS, prolonged survival, or potential to induce autoimmunity have been explored. To determine whether SR CD4^+^ T cells are retained during chronic infection, MOG-specific 2D2 CD4^+^ T cells were transferred to sub-lethally irradiated Wt mice prior to JHMV infection. By enhancing engraftment of donor T cells, this approach increased SR T cells to numbers amenable to flow cytometric analysis, while maintaining a host anti-viral immune response. Bone marrow-derived inflammatory (CD45^hi^) cells were minimal within the CNS of recipients prior to infection (Fig. 1a), indicating non-specific activation and that CNS recruitment was prevented by intact blood brain barrier. At day 7 p.i., maximal anti-viral T cell responses [24, 25] coincided with a decreased percentage of transferred SR T cells in CLN (Fig. 1b, c). Grafted SR T cells were undetectable within the CNS at day 7 p.i. following JHMV infection (Fig. 1b, c) in contrast to their early migration into the CNS during acute MHV-A59 infection [31]. Nevertheless, transferred SR T cells were present in the CNS of JHMV-infected mice by day 14 p.i. (Fig. 1b, c); furthermore, similar proliferation of grafted SR T cells and host CD4^+^ T cells suggested identical activation (Fig. 1d). Although the kinetics differed, these data are consistent with CNS recruitment of SR T cells during MHV-mediated demyelination, independent of the virus strain and tropism [31]. Importantly, retention of transferred SR T cells at slightly declining frequencies within the total CNS CD4 population out to day 30 p.i. (Fig. 1b, c) negated preferential expansion/survival during chronic viral infection. The absolute numbers of grafted SR CD4^+^ T cells gradually declined (Fig. 1c) concomitant with contraction of the overall CD4^+^ T cell population, supporting a lack of ongoing self-Ag-driven survival. Furthermore, retention of SR T cells within the CNS did not alter disease severity out to 30 days p.i. (Fig. 1e). Within the CLN, transferred SR T cells comprised ~40 % of activated CD44^hi^ cells (data not shown) and their absolute numbers remained stable during ongoing chronic JHMV infection (Fig. 1c).

Limited proliferation of SR T cells within the CNS during acute MHV-A59 infection [31] suggested the possibility of non-specific T cell recruitment. To determine the specificity of recruitment into the CNS, heterologous OVA-specific T cells were co-transferred with SR T cells to sub-lethally irradiated mice. Prior to infection, the frequencies of both the SR- and OVA-specific T cells were similar in CLN (Fig. 1f) and blood (data not shown), demonstrating equivalent survival. Following JHMV infection, the relative percentages of both the SR- and OVA-specific T cells were reduced in the CLN coincident with the expansion of virus-specific T cells. However, the decline in transferred SR T cells was less dramatic, suggesting enhanced survival cues via Ag-specific activation (Fig. 1f). Consistent with an absence of non-specific peripheral activation, OVA-specific T cells were not detected in the inflamed CNS at day 14 p.i. By contrast, transferred SR T cells constituted a discrete population (Fig. 1f), confirming migration of peripherally activated cells and supporting Ag-dependent CNS retention of SR CD4^+^ T cells.

### SR T cell activation by CD11b^+^ cells in CLN

**Fig. 2:**
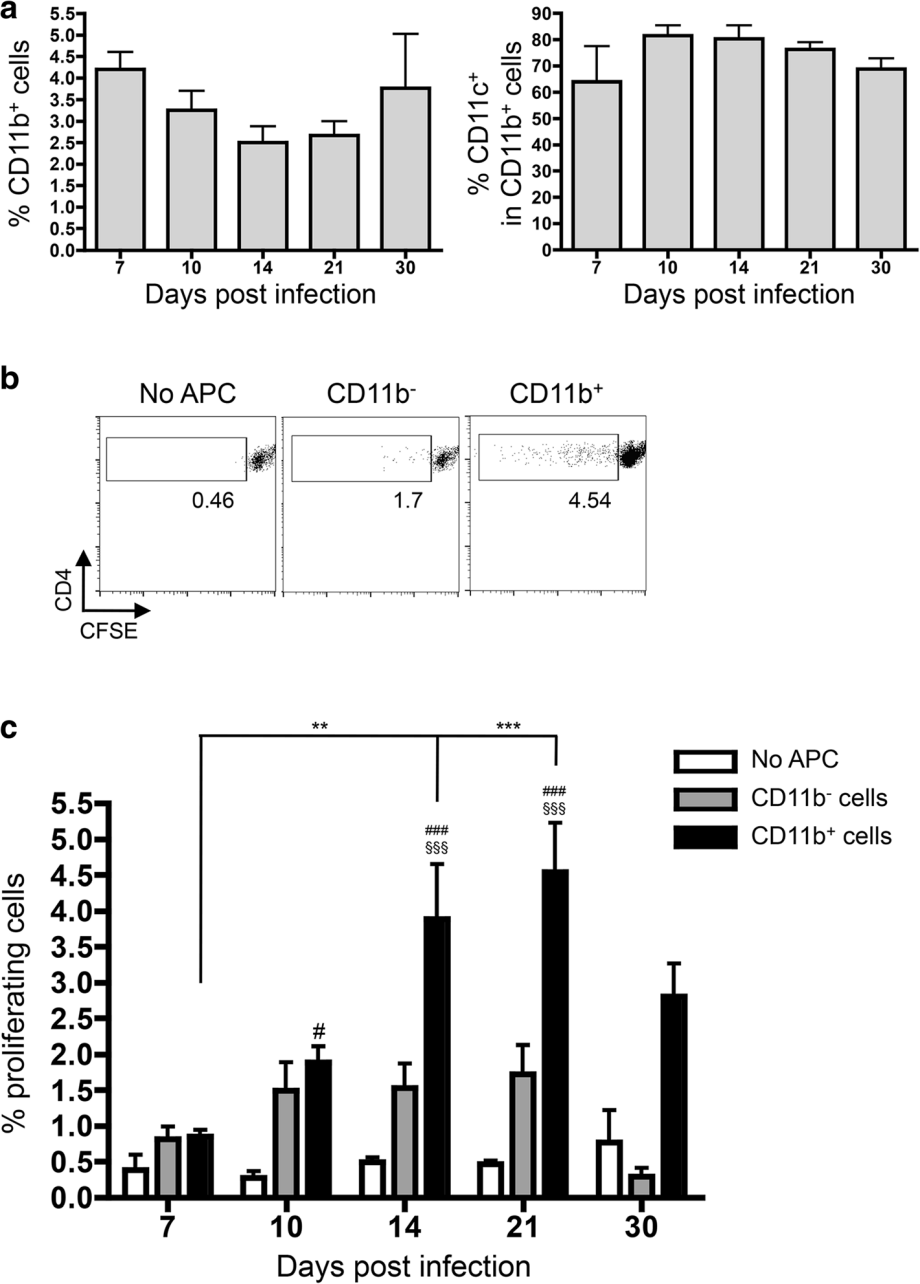
CLN-derived CD11b^+^ cells activate MOG-specific CD4^+^ T cells. **a** Percentages of CD11b^+^ cells and CD11c^+^ cells within the CD11b^+^ population in the CLN from days 7 to 30 p.i. **b** Representative CFSE dilution gated on 2D2 CD90.1^+^CD4^+^ T cells 4 days after co-culture with media only (No APC), CLN-derived CD11b^−^, and CD11b^+^ cells at day 14 p.i. **c** Percentages of proliferating 2D2 cells co-cultured with media only (No APC), CD11b^−^, and CD11b^+^ cells isolated from the CLN of JHMV-infected mice at days 7, 10, 14, 21, and 30 p.i. Data represent the mean ± SEM with *n* = 3–12 pooled mice per time point from three separate experiments. #*p* < 0.05 and ###*p* < 0.001 depict significant differences between No APC and CD11b^+^ cells, whereas §§§*p* < 0.001 depicts significant differences between CD11b^−^ and CD11b^+^ cells determined by a two-way ANOVA with Bonferroni post-test. Significant differences between time points are indicated by **p < 0.01 and ***p < 0.001 using Dunn’s multiple comparison test

CLN constitute the major site of peripheral lymphoid drainage from the CNS [32, 33]. This is supported by activation of JHMV-specific T cells within the CLN [34] and detection of myelin antigens in MS patients and rodents with experimental autoimmune encephalomyelitis (EAE) [35–37], as well as following demyelination induced by oligodendrocyte death [38]. Myelin, either in a soluble form or associated with APC, drains to CLN, where it can potentially activate naïve SR T cells [36, 39] via presentation by CD11b^+^ cells, comprising both dendritic cells (DC) and macrophages [40]. During EAE, the most efficient population-presenting myelin antigen in the CNS is CD11b^+^CD11c^+^ [41, 42]. CD11b^+^ cells represent a small subset (between 2.5 and 4 %) of the CLN population, which only varies slightly throughout the course of JHMV infection (Fig. 2a). Importantly, CD11c^+^ cells constitute the vast majority of CD11b^+^ cells (~70 %) and their frequencies within the CD11b^+^ population remain constant between days 7 and 30 p.i. (Fig. 2a). We therefore tested unfractionated CLN-derived CD11b^+^ cells for the presentation of self-Ag following JHMV infection. CD11b^+^ and control CD11b^−^ cells were purified from the CLN at different times p.i. and were co-cultured with CFSE-labeled MOG-specific CD4^+^ T cells as a source of SR T cells. Neither CD11b^+^ nor CD11b^−^ cells isolated at day 7 p.i. supported SR T cell activation (Fig. 2c). However, by day 10 p.i., when myelin damage becomes apparent, both CD11b^+^ and CD11b^−^ cells initiated minimal T cell proliferation, which reached statistical significance relative to unstimulated conditions (no APC) for CD11b^+^ cells (Fig. 2c). Activation of SR T cells by the CD11b^+^ population increased at days 14 and 21 p.i. concomitant with enhanced demyelination [23, 43], while the CD11b^−^ population retained only a minimal ability to support SR T cell proliferation (Fig. 2b, c). The ability of CLN-derived CD11b^+^ cells to support SR T cell proliferation declined, but was sustained, by day 30 p.i., while the minimal ability of the CD11b^−^ population dropped to below detection by 30 days p.i. (Fig. 2c). The presence of myelin Ag within the CLN by day 14 p.i. supports the possibility that endogenous SR T cells could be activated following JHMV-induced demyelination.

### CNS-infiltrating myeloid cells support SR CD4^+^ T cell responses

**Fig. 3:**
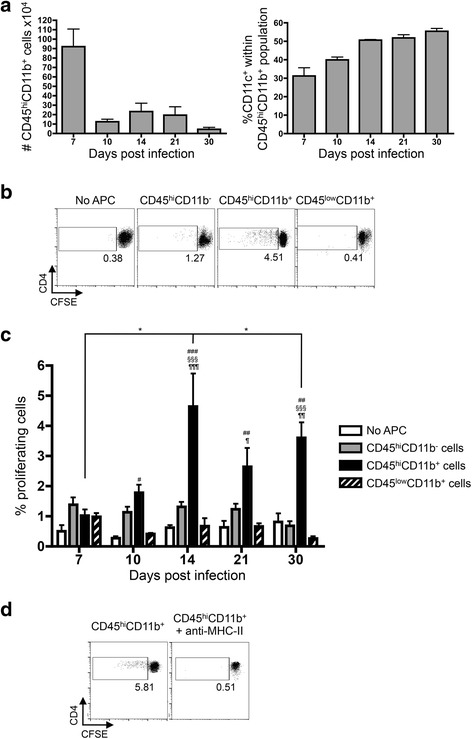
CNS-derived CD45^hi^CD11b^+^ cells present endogenous myelin antigen. **a** Numbers of CD45^hi^CD11b^+^ cells and frequencies of CD11c^+^ cells within CD45^hi^CD11b^+^ populations in the CNS at days 7 to 30 p.i. **b** Representative CFSE dilution gated on 2D2 CD90.1^+^CD4^+^ T cells 4 days after co-culture with media only (No APC), CD45^hi^CD11b^−^, CD45^hi^CD11b^+^ cells, and microglia (CD45^low^CD11b^+^) purified from the CNS at day 14 p.i. **c** Percentages of proliferating 2D2 T cells co-cultured with media only (No APC), CD45^hi^CD11b^−^, CD45^hi^CD11b^+^ and CD45^low^CD11b^+^ cells purified from the CNS between days 7 and 30 p.i. Data represent the mean ± SEM from at least four separate experiments with *n* = 7 pooled mice per time point per experiment. #*p* < 0.05, ##*p* < 0.01, ###*p* < 0.001 depict significant differences between No APC and CD45^hi^CD11b^+^ cells, whereas §§§*p* < 0.001 depicts significant differences between CD45^hi^CD11b^−^ and CD45^hi^CD11b^+^ cells and ¶*p* < 0.05 and ¶¶*p* < 0.01 depict significant differences between CD45^low^CD11b^+^ and CD45^hi^CD11b^+^ cells at a given time point, determined by a two-way ANOVA with Bonferroni post-test. Significant differences between time points are indicated *p < 0.05 using Dunn’s multiple comparison test. **d** Proliferation of 2D2 CD4^+^ T cells co-cultured with CD45^hi^CD11b^+^ isolated from the CNS at day 14 p.i. with or without anti-MHC class II blocking antibody

The absence of clinically apparent autoimmunity, coincident with a decline rather than progressive increase in SR T cells in the CNS during chronic JHMV infection (Fig. 1c), suggested that SR T cells fail to be continuously reactivated within the CNS. In the inflamed CNS, resident microglia and myeloid cells constitute heterogeneous populations of APC potentially capable of presenting self-Ag [40]. To determine whether CNS-infiltrating myeloid cells, comprising macrophages and dendritic cells, and/or microglia are capable of processing and presenting self-Ag within the CNS following infection, both potential APC populations were purified based on differential CD45 expression [44, 45] at distinct times p.i. and tested for the ability to support SR T cell activation in the absence of exogenously added Ag. Infiltrating myeloid cells at day 7 p.i. (Fig. 3a) were comprised of ~30 % CD11c^+^ cells, a proportion that gradually increased during the course of infection to reach ~55 % at day 30 p.i. (Fig. 3a). At the peak of acute inflammation (day 7 p.i.), neither the CD45^hi^CD11b^+^, CD45^hi^CDllb^−^, nor microglia populations supported T cell proliferation (Fig. 3c). However, SR T cell proliferation was modestly increased by the CD11b^+^ APC subset at day 10 p.i. (Fig. 3c) and prominently by day 14 p.i. (Fig. 3b, c), similar to the CLN (Fig. 2). On a per cell basis, the ability of infiltrating CD11b^+^ cells to support proliferation remained relatively stable during chronic JHMV infection (Fig. 3c). Although CD11b^−^ cells appeared to promote minimal proliferation, their activity remained near threshold levels throughout the infection. Similar to the CD11b^−^ population, microglia were unable to support SR T cell proliferation at any time p.i. (Fig. 3b, c). The kinetics of SR CD4^+^ T cell activation by CNS-infiltrating CD11b^+^ cells correlate with viral-induced myelin destruction as a source of self-Ag [43]. In addition, anti-MHC class II Ab blocked proliferation, supporting MHC class II-dependent T cell activation (Fig. 3d) rather than non-specific proliferation signals derived from the inflamed CNS environment (Fig. 1d).

**Fig. 4:**
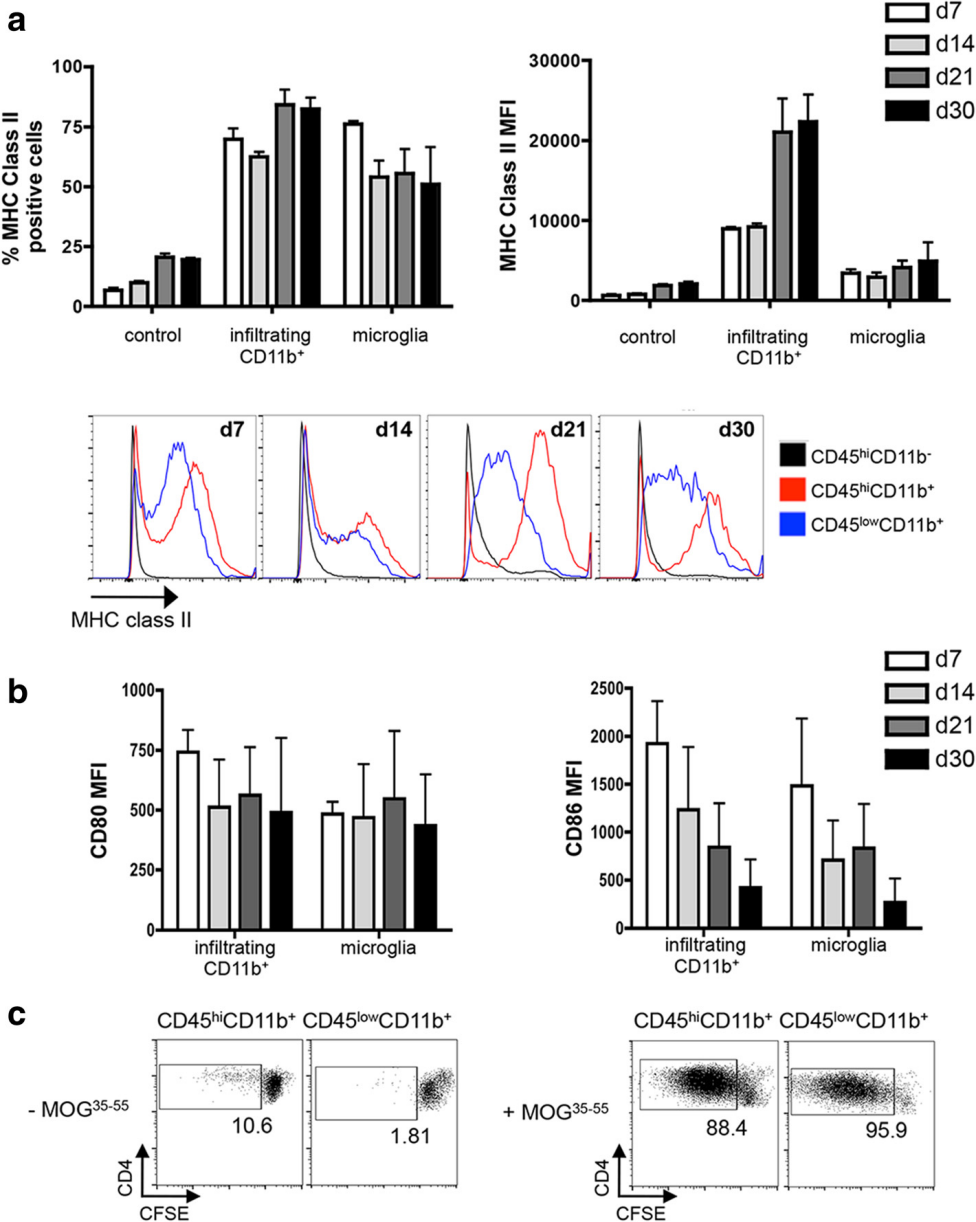
MHC class II expression and exogenous myelin antigen presentation by CNS-infiltrating CD11b^+^ cells and microglia. **a** Percentage and mean fluorescence intensity (MFI) expression of MHC class II by CNS-derived control (CD45^hi^CD11b^−^), infiltrating CD11b^+^ (CD45^hi^CD11b^+^), and microglia (CD45^low^CD11b^+^) cells between days 7 and 30 p.i. Data represent the average ± SEM of three separate experiments with *n* = 3 pooled mice per experiment. Histogram of MHC class II expression by CD45^hi^CD11b^−^ (*black line*), infiltrating CD11b^+^ (*red line*), and microglia (*blue line*) during JHMV infection. **b** MFI expression of CD80 and CD86 by CNS-infiltrating CD11b^+^ (CD45^hi^CD11b^+^) and microglia (CD45^low^CD11b^+^) cells between days 7 and 30 p.i. Data represent the average ± SEM of 3 separate experiments with n = 3 pooled mice per experiment. **c** Proliferation of 2D2 CD4^+^ T cells co-cultured with CD45^hi^CD11b^+^ and CD45^low^CD11b^+^ cells with or without exogenously added MOG^35-55^ peptide

MHC class II expression was compared on microglia and infiltrating CD11b^+^ cells by flow cytometry to examine whether the inability of microglia to support T cell proliferation correlated with differential MHC class II expression. While no differences were noted in the percentage of class II-expressing cells within the populations (Fig. 4a), microglia were inferior to infiltrating CD11b^+^ cells in their level of class II expression based on MHC class II mean fluorescence intensity (MFI) (Fig. 4a), even at the peak of IFN-γ release at day 7 p.i. [25]. Similarly, the MFI of the co-stimulatory molecule CD86 was slightly lower on microglia compared to infiltrating CD11b^+^ cells, while no differences were observed for CD80 expression levels (Fig. 4b). Moreover, while MHC class II expression was sustained at similar levels on microglia, it was further upregulated between day 14 and 21 p.i. on infiltrating CD11b^+^ cells (Fig. 4a). To assess whether myelin Ag added to the cell culture could overcome the inability of microglia to activate SR T cells, CD11b^+^ APC populations were pre-incubated with MOG peptide. Under these conditions, microglia induced proliferation of SR CD4^+^ T cells as well as infiltrating CD11b^+^ cells (Fig. 4c). These data demonstrate that while class II levels on microglia suffice to present exogenous myelin peptide to activate cognate T cells, only infiltrating CD11b^+^ cells act as APC of endogenous myelin Ag to SR T cells; importantly, this capacity is retained throughout chronic infection.

**Fig. 5:**
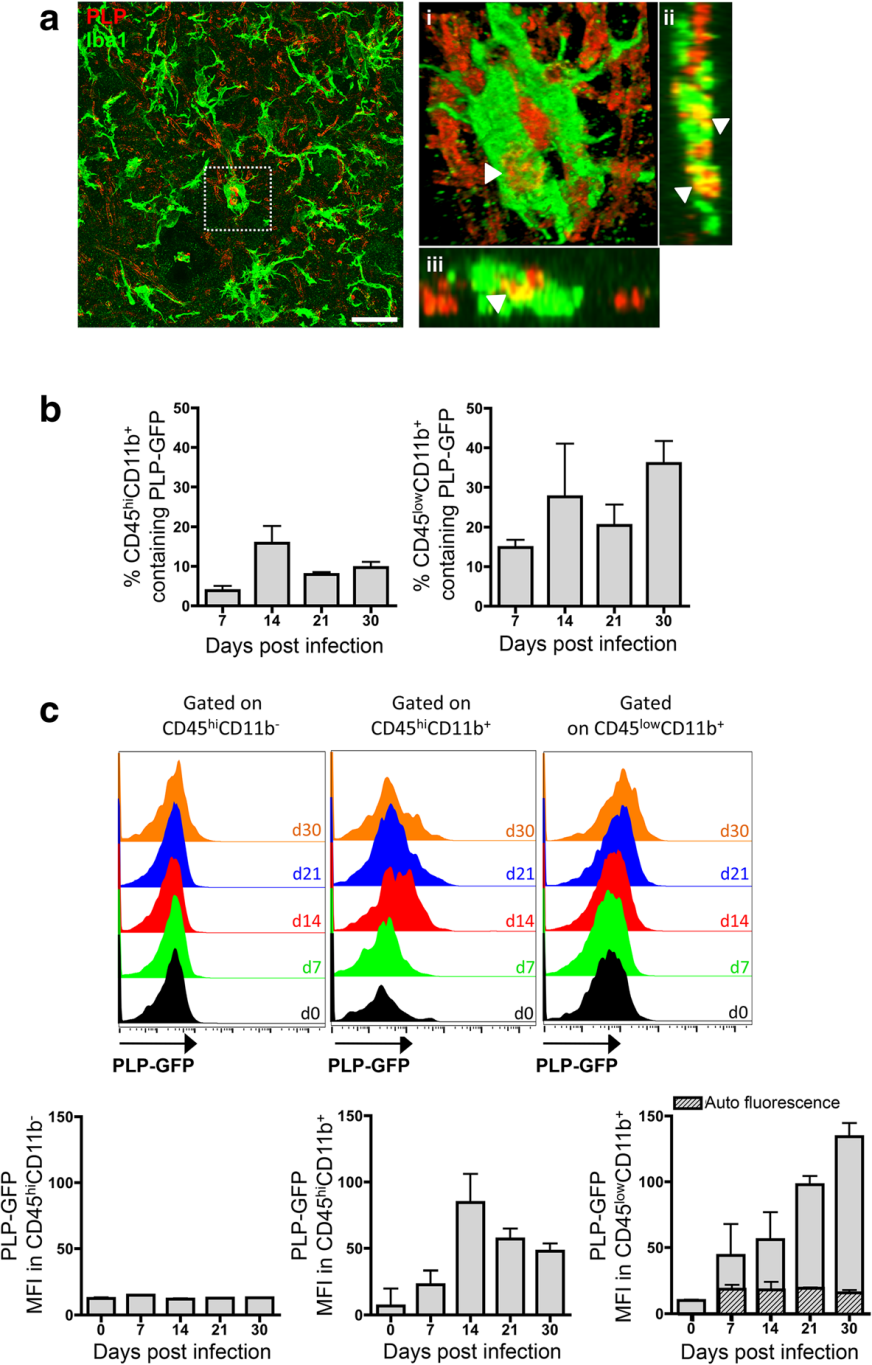
Myelin debris within CNS cells. **a** PLP (*red*) and Iba1 (*green*) stainings on spinal cord tissue isolated from Wt-infected mice at day 21 p.i. and analyzed by confocal microscopy. Scale bar, 10 μm. (*i*) 3D reconstruction image view, *yz* (*ii*), and *xz* (*iii*) projections show PLP^+^ myelin in close contact and engulfed (*arrow head*) by Iba1^+^ cells. **b** Percentage of CD45^hi^CD11b^+^ and microglia (CD45^low^CD11b^+^) containing GFP between days 7 and 30 p.i. during infection of PLP^GFP^ mice. **c** Mean GFP fluorescent intensity (MFI) analyzed by flow cytometry in CNS-infiltrating CD45^hi^CD11b^−^ and CD45^hi^CD11b^+^ cells and microglia (CD45^low^CD11b^+^) between days 0 to 30 p.i. *Hashed bars* represent microglia auto-fluorescence detected within infected Wt mice between days 7 and 30 p.i. Data represent the mean ± SEM from two separate experiments with *n* = 3 per time point per experiment

The inability of microglia to support SR T cell proliferation may reflect a defect in myelin uptake or Ag processing. Myelin engulfment by Iba1^+^ cells supports myelin uptake by microglia and/or infiltrating myeloid cells during JHMV-induced demyelination (Fig. 5a). However, the amoeboid morphology of microglia during inflammation prevents histological distinction between myelin in macrophages versus microglia (Fig. 5a). Mice expressing GFP under the PLP promoter were therefore infected to quantify GFP as a surrogate marker for myelin ingestion by infiltrating CD11b^+^ cells versus microglia. Flow cytometry revealed GFP within both infiltrating myeloid cells (CD45^hi^CD11b^+^) and microglia (CD45^low^CD11b^+^) (Fig. 5b, c). Percentages of infiltrating myeloid cells and microglia containing GFP were relatively stable between days 7 and 30 p.i. (Fig. 5b), with a higher proportion of microglia containing GFP, supporting their primary role in demyelination [46]. In contrast to percentages, MFI analysis showed that the amount of GFP differed throughout the course of infection (Fig. 5c). Infiltrating CD11b^+^ cells exhibited peak uptake at 14 days p.i., which slowly decreased at later time points (Fig. 5c), but remained higher than GFP MFI in the control CD45^hi^CD11b^−^ population, as well as CD45^hi^CD11b^+^ cells isolated from naïve animals (Fig. 5c). By contrast, a gradual increase in GFP was observed within microglia throughout the course of JHMV infection independent of auto-fluorescence (Fig. 5c). These data imply that both the infiltrating myeloid and microglial populations phagocytoze myelin during viral-induced demyelination. Therefore, the inability of microglia to support SR T cell activation is less likely to reside in a defect in myelin uptake, rather than inefficient Ag processing and/or presentation.

### SR T cells within the CNS during chronic viral infection

**Fig. 6:**
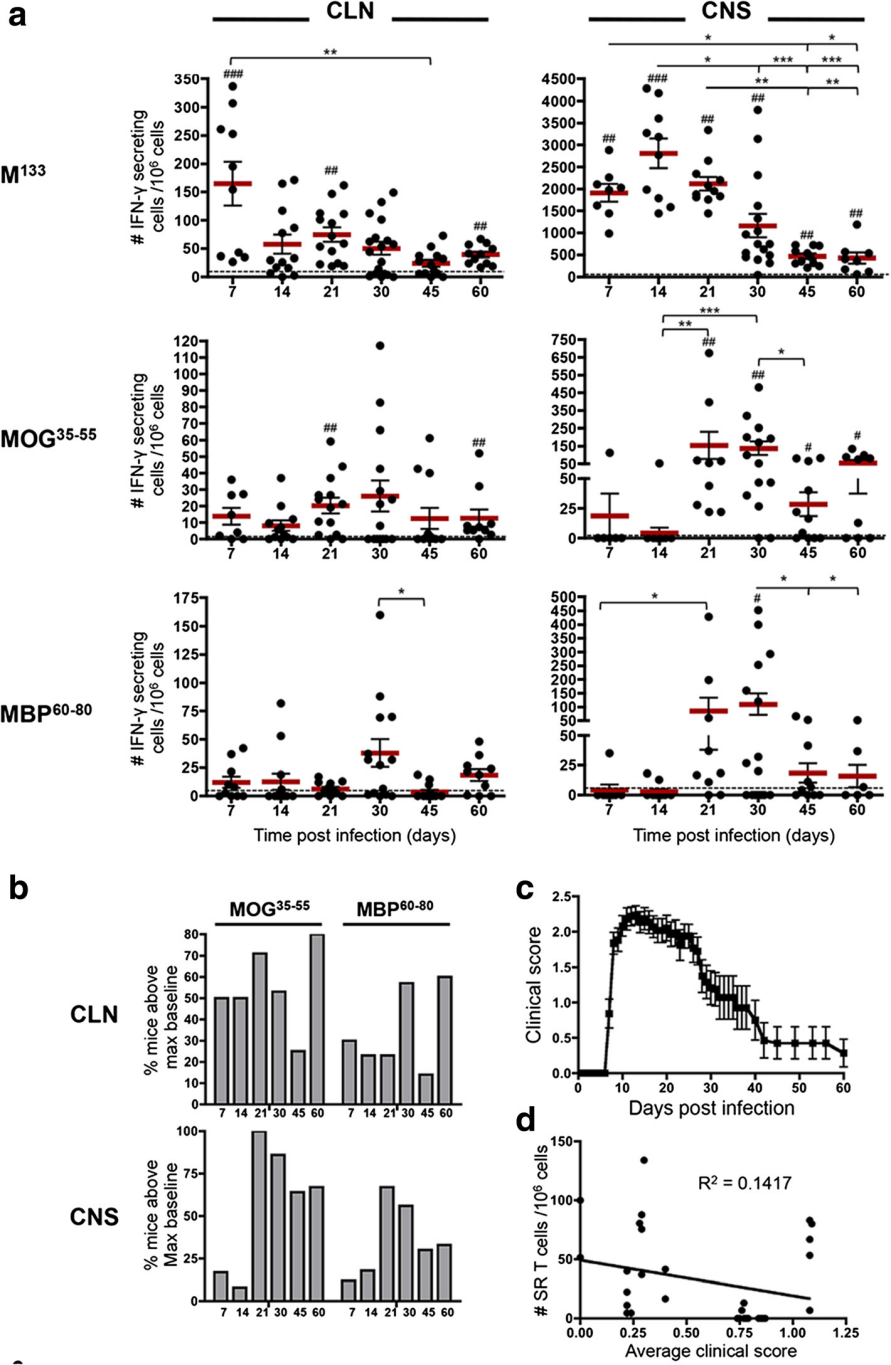
Myelin-specific CD4^+^ T cell activation following JHMV infection. **a** Number of virus-specific M^133^, MOG^35-55^-specific, and MBP^60-80^-specific CD4^+^ T cells per 10^6^ cells in the CLN and CNS of JHMV-infected mice between days 7 to 60 p.i. Each *dot* represents the triplicates of individual experiments at various dilutions of at least eight pooled mice. *Dashed lines* represent the limit of detection determined as frequencies of IFN-γ-secreting cells specific for M^133^, MOG^35-55^, and MBP^60-80^ detected in naïve animals. *Red bars* represent the mean ± SEM. Significant differences between time points are indicated *p < 0.05, **p < 0.01, and ***p < 0.001. Significant differences compared to baseline levels are indicated #*p* < 0.05, ##*p* < 0.01, and ###*p* < 0.001 using unpaired *t* test. **b** Percentage of mice exhibiting IFN-γ-secreting MOG and MBP-specific CD4^+^ T cells above maximal baseline levels in the CLN and CNS between days 7 and 60 p.i. **c** Clinical symptoms following JHMV infection. Data represent the mean ± SEM of 32 mice. **d** Correlation between frequencies of SR T cells at days 45 and 60 p.i. with average clinical scores, coefficient of determination *R* ^2^ = 0.1417

Myelin damage, uptake and subsequent presentation of self-Ag by potential APC, and sustained CNS inflammation without overt clinical evidence of autoimmune disease following JHMV infection pose a dilemma. Plausible explanations may be that SR T cells are either not, or only minimally, induced or that they are not reactivated within the CNS. Indeed, demyelination triggered by oligodendrocyte death fails to initiate autoimmunity, despite myelin Ag drainage to CLN [38]. To determine if JHMV-induced demyelination results in the activation of endogenous SR T cells, CD4^+^ T cell responses to the H-2^b^ restricted encephalitogenic myelin epitopes MOG^35-55^ and MBP^60-80^ were assessed by Enzyme-Linked ImmunoSpot (ELISPOT) to detect low-frequency responder cells. Responses to the H-2^b^-restricted immuno-dominant viral M^133^ epitope [47] were used as positive controls. Analysis focused on IFN-γ-producing CD4^+^ T cells, as JHMV infection induces a vigorous Th1 response with negligible IL-17 or IL-9 components (data not shown), which is supported by no affects on pathogenesis in the absence of IL-23 [48]. Virus- as well as myelin-specific T cell frequencies were lower than 9 and 5 positive cells per 10^6^, respectively, within the CLN and spleen of naïve animals (Fig. 6a). Within CLN, the frequency of virus-specific CD4^+^ T cells peaked at day 7 p.i., declined by day 14 p.i., and remained relatively stable up to day 60 p.i. (Fig. 6a). Within the CNS, frequencies of virus-specific T cells were ~10 higher than CLN at day 7 p.i., continued to increase at day 14 p.i., and declined as the overall inflammatory response resolved due to virus control (Fig. 6a).

SR T cells specific for both the immuno-dominant MOG and myelin basic protein (MBP) epitopes within the CLN were above baseline throughout acute and chronic JHMV infection, albeit at very low frequencies (Fig. 6a). Maximum SR T cell frequencies were observed at day 30 p.i. (Fig. 6a) with responses near detection limits in a large proportion of infected mice throughout infection (Fig. 6b). A very limited number of infected mice harbored SR T cells within the CNS during acute infection at days 7 and 14 p.i. However, by day 21 p.i., the number of responder mice exhibiting SR T cells within the CNS reached 100 % (Fig. 6b), coincident with peak frequencies of both MOG- and MBP-specific T cells between days 21 and 30 p.i. (Fig. 6a). These data are consistent with maximal clinical disease throughout days 14–21 p.i. in all infected mice (Fig. 6c). Although the SR T cell frequencies in the CNS were variable among individual mice, and always reduced relative to virus-specific T cells, they were increased relative to the CLN at day 21 p.i. and thereafter (Fig. 6a). Importantly, CNS SR T cells were sustained above baseline during resolution of clinical symptoms, with preferential stability of MOG-specific T cells (Fig. 6a, b). However, compared to peak SR T cell frequencies at days 21/30 p.i., frequencies, as well as the number of mice harboring CNS SR T cells, declined as clinical disease resolved (Fig. 6c). These data demonstrate that endogenous SR T cells are induced in the vast majority of JHMV-infected mice. However, they are only sustained within the CNS of ~60 % of mice during chronic demyelination, when lesion formation is counterbalanced by repair (Fig. 6a, b). Importantly, segregation of mice harboring SR T cells within the CNS after day 30 versus mice in which SR T cells were undetectable showed no differences in overall clinical recovery (Fig. 6d). These data further supported our previous observations that CNS recruitment and retention of SR T cells did not alter disease severity (Fig. 1e).

## Discussion

Animal models have demonstrated that viral infections can promote the initiation as well as increase the severity of autoimmune diseases [4]. However, evidence for a viral etiology of human autoimmune diseases, including MS, remains elusive. Moreover, given the extent of both acute and persisting viral infections without evidence of autoimmune sequelae, it has also been proposed that infections may even provide protection from autoimmunity. Specifically, the conditions promoting or stemming activation of auto-Ag-specific T cells following viral-induced tissue damage have not been extensively explored. Chronic CNS infection by the naturally occurring mouse pathogen TMEV promotes activation of SR T cells, which mediates increasing tissue destruction accompanied by an ascending paralysis [20]. By contrast, infection with JHMV also leads to immune-mediated demyelination; however, persistent infection correlates with clinical recovery [27]. While clinical improvement throughout persistence despite ongoing demyelination supported the absence of an autoimmune component following neurotropic MHV infection, previous studies clearly demonstrate sufficient auto-Ag presentation to induce SR T cell responses [31, 49, 50]. In an effort to reconcile peripheral activation of SR T cells, but no disease exacerbation, this study set out to assess kinetics of auto-Ag presentation as well as the fate of SR T cells following JHMV infection. The data demonstrate the presence of APC capable of activating SR T cells in both the CLN and the CNS with maximal stimulating activity during the time of most overt demyelination. While both the CNS-infiltrating myeloid population and microglia ingest GFP as a surrogate for myelin, only the CNS-infiltrating APC were capable of presenting endogenous myelin Ag to SR T cells ex vivo. Finally, the results demonstrate that SR T cells are activated from the endogenous T cell repertoire when myelin destruction is evident, but infectious virus is already controlled. Although SR T cells migrate into and are most prominent in the persistently infected CNS during maximal demyelination, no evidence was found to suggest preferential expansion despite ongoing demyelination, consistent with minimal re-stimulation in vivo.

Mechanisms underlying SR T cell activation following CNS infections include molecular mimicry between pathogen and self-epitopes, presentation of self-peptides from tissue damage, and dysregulation of regulatory tolerance mechanisms [51, 52]. Myelin-specific T cells isolated from MS patients have been shown to cross-react with human coronavirus [53, 54]. Similarly, homology was found between MHV and myelin peptides, potentially triggering autoimmune disease [55]. Nevertheless, SR T cells were near detection levels within the CNS during acute JHMV infection in the vast majority of mice and their frequency only increased as myelin damage increased. Moreover, their numbers declined as clinical symptoms dropped due to increased repair. Self-Ag-specific activation of SR T cells is further supported by the lack of T cell activation with irrelevant specificity and blockade of activation by anti class II Ab, ruling out bystander events. Overall, our data support the hypothesis that release of myelin debris due to JHMV-induced tissue damage results in cell-free or cell-associated myelin drainage to CLN, where a population of CD11b^+^ APC can support the activation of SR T cells. Activated SR T cells access the CNS at a time when the vast majority of infectious virus is already controlled, but persistence drives ongoing expression of proinflammatory molecules capable of recruiting activated cells from circulation [56]. Local encounter with myelin-loaded CD45^hi^CD11b^+^ APC, but not microglia, is then poised to drive SR T cell reactivation. The apparent inability of microglia to activate SR CD4^+^ T cells ex vivo remains unclear as their uptake of myelin is consistent with demyelination independent of inflammatory monocyte recruitment following JHMV infection [46]. Nevertheless, microglia also failed to support CD4^+^ T cell responses following TMEV infection and induction of EAE [57, 58]. In fact, several studies question the role of microglia as APC in vivo [59] despite their implication as APC based on expression of MHC class II and co-stimulatory molecules in both MS patients [60] and EAE [61], as well as their ability to activate CD4^+^ T cells in vitro [57]. Detection of myelin within Iba1^+^ cells increased GFP MFI in microglia, as well as detection of myelin in microglia in other models [57, 58] supports self-Ag uptake during JHMV infection. However, these data suggest that microglia may be poor at processing myelin Ag for loading onto MHC class II. This notion is also supported by effective priming of SR T cells using exogenous peptide, despite reduced MHC class II expression relative to infiltrating CD11b^+^ cells. In addition, as activated microglia can adapt to their surroundings to mediate both pro- and anti-inflammatory functions [62], it cannot be excluded that microglia may negatively regulate SR T cell responses during chronic JHMV infection by limiting T cell proliferation or by inducing apoptosis [63].

The results also indicate a discrepancy between APC presentation capacity ex vivo, yet apparently limited SR T cell activation in vivo. Although APC in CLN sustain their ability to activate SR T cells throughout days 14 to 30 p.i., endogenous SR T cells are only detected at low frequencies and exhibit a brief peak at day 30 p.i. Maximal accumulation of SR T cells in the CNS between days 21 and 30 p.i. in all mice supports efficient egress from CLN. Whether frequencies in the CNS are supported by ongoing recruitment or local expansion remains unclear. The capacity of CNS-infiltrating CD11b^+^ cells to activate SR T cells ex vivo in the absence of exogenous peptide indicates that APC are competent to reactivate SR T cells throughout chronic infection. However, no evidence for preferential expansion or retention of SR T within the CNS relative to virus-specific CD4^+^ T cells suggests that the absence of sustained activation may contribute to the lack of increasing clinical symptoms during chronic infection. These results clearly differ from the TMEV model, in which SR T cells aggravate pathology and clinical disease. One possible scenario is that persistent JHMV infection may establish an environment that suppresses the SR T cell response. JHMV infection induces both Ag-specific and Foxp3^+^ regulatory T cells [64, 65] which are efficient at controlling autoimmunity in other settings [66, 67]. Treg depletion [65], elimination of Ag-specific Treg [65], and adoptive transfer of Foxp3^+^ Treg [68] during acute JHMV infection all demonstrated that, in contrast to TMEV [69], Tregs have a limited effect on virus clearance. Nevertheless, Foxp3^+^ Tregs play a critical role in limiting tissue destruction [68], suggesting that they regulate the activation or effector function of SR T cells following JHMV-induced demyelination [70]. Although Treg control of SR T cells in vivo and their association with increased tissue destruction remains unknown, depletion of Treg correlates with increased proliferation of SR T cells in the CLN during acute MHV-A59 infection [31]. Whether Tregs act in limiting SR T cell induction during JHMV infection, which does not or only poorly replicates in CLN, or whether additional factors limit SR T cell responses at the target site remains to be elucidated.

## Conclusions

This study demonstrates the activation and kinetics of SR CD4^+^ T cells within the endogenous T cell repertoire relative to self-Ag presentation by APC following JHMV-induced demyelination. Importantly, ongoing myelin loss, sustained CNS-derived APC activity ex vivo, and retention of SR T cells do not lead to autoimmune disease during chronic JHMV infection. This model thus provides a unique opportunity to determine mechanisms preventing autoimmunity in the context of a persistent viral infection.

## Abbreviations

APC: antigen-presenting cell
BrdU: bromodeoxyuridine
CFSE: carboxyfluorescein succinimidyl ester
CLN: cervical lymph node
CNS: central nervous system
DC: dendritic cells
GFP: green fluorescent protein
IFN-γ: interferon-γ
MBP: myelin basic protein
MFI: mean fluorescence intensity
MHC: major histocompatibility complex
MHV: mouse hepatitis virus
MOG: myelin oligodendrocyte glycoprotein
MS: multiple sclerosis
OVA: ovalbumine
PFA: paraformaldehyde
PLP: proteolipid protein
SR: self-reactive
TCR: T cell receptor
TMEV: Theiler’s murine encephalomyelitis virus
Wt: wild type
